# Multiple target detection using photonic radar for autonomous vehicles under atmospheric rain conditions

**DOI:** 10.1371/journal.pone.0322693

**Published:** 2025-05-13

**Authors:** Sushank Chaudhary, Sunita Khichar, Yahui Meng, Abhishek Sharma

**Affiliations:** 1 School of Computer, Guangdong University of Petrochemical Technology, Maoming, China; 2 Department of Electrical Engineering, Chulalongkorn University, Bangkok, Thailand; 3 School of Science, Guangdong University of Petrochemical Technology, Maoming, China; 4 Department of Electronics and Communication Engineering, National Institute of Technology, Hamirpur, Himachal Pradesh, India; Parul University, INDIA

## Abstract

Photonic radar systems offer a promising solution for high-precision sensing in various applications, particularly in autonomous vehicles, where reliable detection of obstacles in real-time is critical for safety. However, environmental conditions such as atmospheric turbulence and rain attenuation significantly impact radar performance, potentially compromising detection accuracy. This study aims to assess the performance of a photonic radar system under different environmental scenarios, including free-space, Gamma-Gamma atmospheric turbulence, and light and heavy rain conditions, with a focus on detecting three distinct targets positioned at various distances. Our simulations demonstrate that Gamma-Gamma atmospheric turbulence introduces variability in the received signal, with fluctuations becoming more pronounced at greater distances. Additionally, rain attenuation was found to substantially degrade performance, with heavy rain causing up to a 1 dBm reduction in received power at 50 meters and nearly a 1.5 dBm reduction at 100 meters, compared to light rain. For three targets located at 50m, 100m, and 150m, the combined effects of rain and turbulence were particularly noticeable at longer distances, with the received power under heavy rain dropping to −100.4 dBm at 150 meters. These findings indicate the importance of accounting for environmental conditions in the design of photonic radar systems, especially for autonomous vehicle applications. Future improvements could focus on developing adaptive radar techniques to compensate for adverse weather effects, ensuring robust and reliable performance under varying operational conditions. The novelty of this study lies in the integration of photonic radar technology with an advanced modeling framework that accounts for both free-space propagation and adverse weather conditions. Unlike conventional radar studies, our work incorporates Gamma-Gamma turbulence modeling and rain attenuation effects to provide a more comprehensive analysis of radar performance in real-world environments. This study also proposes an optimized detection strategy for multiple targets at varying distances, demonstrating the potential of photonic radar for autonomous vehicle applications.

## 1 Introduction

**Fig 1:**
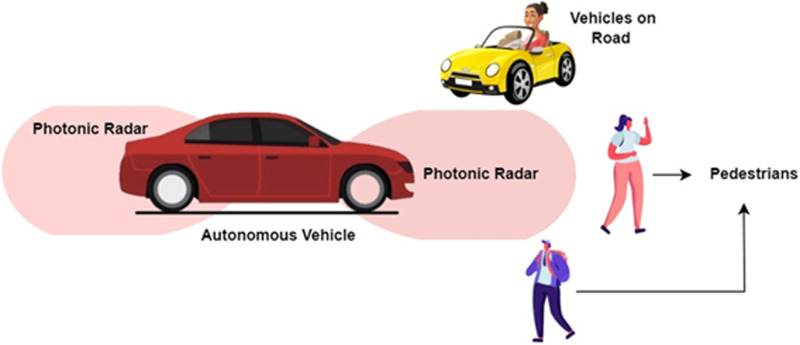
Photonic radar equipped autonomous vehicle.

Autonomous vehicles are at the forefront of the next transportation revolution, promising to reduce accidents, optimize traffic flow, and increase convenience for passengers [1–3]. However, one of the key technological challenges preventing widespread deployment is the need for reliable and high-performance sensing systems [4,5]. Autonomous vehicles must operate in a variety of environments, from urban streets with dense traffic to rural roads with minimal infrastructure. This requires robust object detection and environmental awareness systems that can accurately perceive and track multiple objects in real-time, regardless of weather conditions or the complexity of the surroundings [6,7]. Currently, a typical autonomous vehicle employs a combination of sensors such as cameras, LiDAR (Light Detection and Ranging) [8–10], ultrasonic sensors, and traditional radar to gather information about its environment. While this sensor fusion approach provides useful data, each sensor type has its own limitations. Cameras, for instance, are heavily dependent on lighting conditions and can struggle in low-light or foggy environments, significantly affecting their ability to detect objects accurately [11]. They also have difficulty providing reliable-deep information, which is critical for tasks such as obstacle avoidance or precise lane-keeping. LiDAR, while offering high-resolution 3D mapping and accurate distance measurements, also has its limitations [12–14]. It tends to be sensitive to environmental interference such as heavy rain, fog, or snow, and its operational range is relatively short, limiting its effectiveness at high speeds or in open-road conditions. Moreover, the high cost and complexity of LiDAR systems make them less ideal for widespread commercial use in autonomous vehicles [15,16]. Traditional radar, on the other hand, has proven effective in detecting objects over longer distances and in poor visibility conditions, such as rain, snow, or fog [17]. However, conventional radar technology is constrained by limited bandwidth, which reduces its ability to provide fine spatial resolution [18]. This results in a reduced capacity to accurately differentiate between closely spaced objects, such as a pedestrian walking next to a parked vehicle. Additionally, traditional radar systems are vulnerable to electromagnetic interference, which can reduce their reliability in environments with high signal congestion, such as urban areas with many overlapping radar signals from other vehicles or infrastructure [19]. To address these limitations, a new sensing technology is needed—one that offers both high resolution and precision, while being resilient to environmental conditions and interference. This is where photonic radar emerges as a compelling solution [20,21]. Fig 1 shows the photonic radar equipped autonomous vehicle. Photonic radar leverages the principles of photonics, which involve the use of light (photons) to process and transmit information. Unlike traditional radar, which relies on electronic signal processing, photonic radar uses optical signals, offering several key advantages for autonomous vehicle sensing. Firstly, photonic radar can operate over significantly wider bandwidths compared to conventional radar systems. This increased bandwidth translates to finer resolution, enabling the system to detect and differentiate between small, closely spaced objects with high accuracy [22].

This is particularly important in urban driving environments where vehicles need to navigate around pedestrians, cyclists, and other vehicles in tight spaces. Secondly, photonic radar systems are largely immune to electromagnetic interference. This resilience makes them highly suitable for operation in crowded electromagnetic environments, such as cities where multiple vehicles and infrastructure components may be using radar systems simultaneously [23]. Moreover, the use of light-based signals allows photonic radar to function effectively in adverse weather conditions, such as heavy rain, snow, or fog—scenarios where cameras and LiDAR often struggle [24]. In addition to its environmental robustness, photonic radar has the potential to enhance the overall safety and performance of autonomous vehicles by providing high-resolution data in real-time [25]. The optical components used in photonic radar systems are capable of processing data at the speed of light, allowing for faster decision-making and more responsive control systems [26]. This rapid processing capability is critical for autonomous driving, where decisions often need to be made within fractions of a second to ensure the safety of passengers and other road users. The integration of photonic radar into autonomous vehicles offers a future where these vehicles can navigate with greater confidence and precision [27]. In this work, we focus on the performance analysis of a photonic radar system designed for use in autonomous vehicles, where reliable and accurate obstacle detection is crucial for operational safety. The primary aim is to assess how environmental factors, specifically atmospheric turbulence and rain attenuation, impact the performance of photonic radar. The novelty of this work lies in its comprehensive approach to modeling photonic radar performance under diverse environmental conditions. Unlike prior studies that focus on either free-space models or simplistic attenuation effects, we integrate Gamma-Gamma turbulence modeling and rain attenuation analysis to offer a more realistic evaluation of photonic radar performance. Additionally, this study proposes an optimized multi-target detection strategy that enhances detection accuracy and range estimation in adverse weather conditions. These contributions demonstrate the potential of photonic radar as a superior sensing technology for autonomous vehicles operating in real-world scenarios. The main contributions in this work are mentioned as following:

1. **Modeling of Photonic Radar in Free-Space and Turbulent Environments:** We present a detailed simulation of photonic radar under free-space conditions and incorporate the Gamma-Gamma fading model to represent atmospheric turbulence. This model accounts for both large-scale and small-scale fading due to fluctuations in the refractive index of the atmosphere.
2. **Impact of Rain Attenuation:** We evaluate the radar system’s performance under light and heavy rain conditions, using rain attenuation models to analyze signal degradation. The effect of rain intensity on received power is quantified, showing significant degradation as rain intensity increases, particularly at longer distances.
3. **Comparison of Environmental Effects:** A comparative analysis is provided between the free-space model and the Gamma-Gamma channel model, highlighting the variability introduced by atmospheric turbulence. Additionally, the performance under light and heavy rain is compared for different target distances, providing insights into the effects of rain on radar signal propagation.
4. **Target Detection and Range Estimation:** We simulate and visualize the radar system’s ability to detect multiple targets at different distances (50m, 100m, and 150m). The results demonstrate how the system’s performance degrades with distance and under adverse environmental conditions.
5. **Simulation-Based Insights for Autonomous Vehicle Applications:** We demonstrate the practical implications of the findings for autonomous vehicles, emphasizing the need to account for atmospheric conditions and rain attenuation in radar system design to ensure robust and reliable detection in real-world scenarios.

## 2 Related works

Photonic radar systems have shown significant progress in recent years, becoming crucial for various applications, including autonomous vehicles and high-resolution imaging. This section reviews advancements in photonic radar technologies, particularly focusing on research from 2020 to 2024 that addresses challenges such as weather resilience, dual-target detection, and filtering techniques. In 2020 [28], the authors of a key study proposed a chip-based microwave-photonic radar for high-resolution imaging. The system, built on a silicon photonic platform, integrated both wideband signal generation and a de-chirp receiver on a single chip, covering the full Ku band (12–18 GHz). This radar demonstrated high-precision distance measurement with a resolution of 2.7 cm, and its compact form factor made it ideal for applications in unmanned aerial vehicles (UAVs) and autonomous vehicles. Building on these developments, in 2021 [29], the authors introduced an advanced photonics-based radar signal generation technology. This study focused on practical implementations of microwave photonic systems in radar applications, addressing challenges such as large modulation bandwidth, tunable pulse duration, and enhanced coherence. By employing optical phase-locked loops and dual electro-optic frequency combs, the system was able to generate reconfigurable radar signals with high precision, making it suitable for next-generation radar systems with a low probability of interception. Further enhancing the field, in another 2021 study [30], the authors conducted a field experiment using an X-band photonic radar for low-RCS target detection and high-resolution image acquisition. The system used photonic frequency quadrupling for signal transmission and a delay interferometer with a balanced photo-detector to improve the signal-to-noise ratio (SNR). This system successfully detected a commercial hexacopter drone from 2.7 km away and captured high-resolution images when the drone was hovering 1.1 km from the radar site. In another work [31], a different approach was taken with the development of a MIMO-employed coherent photonic radar (MIMO-Co-PHRAD), using a linear frequency-modulated continuous-wave technique. This radar system was tested under varying atmospheric conditions such as fog and rain and demonstrated a significant improvement in detection range and SNR compared to single-input single-output radar (SISO-Co-PHRAD). The use of spatial diversity in photonic radar systems was highlighted, offering enhanced performance for applications such as autonomous vehicles. In 2022 [32], the focus shifted towards overcoming bandwidth limitations and electromagnetic interference with the introduction of a microwave photonic radar utilizing sparse stepped frequency chirp (SSFC) signals. This radar system achieved high-resolution detection and anti-interference capabilities by generating a sparse spectrum spanning 18 GHz but occupying only a 4.5 GHz effective spectrum. The radar successfully distinguished two simulated point targets with a distance of 8.3 mm, highlighting its high precision and imaging capabilities. In another 2022 study [33], the authors developed an 11-GHz bandwidth photonic radar using MHz electronics. This system achieved centimeter-level spatial resolution and demonstrated a real-time imaging rate of 200 frames per second. The use of low-speed electronics for signal generation and processing reduced hardware complexity, making the system suitable for high-resolution detection of moving objects like UAVs in applications such as autonomous driving and environmental surveillance. Moving into 2023 [34], the authors proposed a broadband photonic radar with coherent receiving for high-resolution detection. Using frequency quadrupling based on a dual-parallel Mach-Zehnder modulator (DPMZM), the radar achieved an 8 GHz bandwidth, leading to a resolution better than 2 cm. The system’s balanced photonic I/Q de-chirping suppressed common-mode interference and image frequencies, making it highly suitable for applications requiring interference-suppressed, high-resolution detection, such as autonomous vehicles. Later in 2023 [35], another study analyzed the performance of a coherent frequency-modulated continuous-wave (FMCW) photonic radar system under various atmospheric conditions, including solar noise. This research highlighted the radar’s performance in clear, hazy, and foggy weather conditions, demonstrating the degradation in SNR caused by solar noise and different weather conditions. These results emphasized the system’s potential for real-time applications in autonomous vehicles. Most recently, in 2024 [36], the authors proposed a Photonics-Based MIMO Radar with Broadband Digital Coincidence Imaging (DCI). The radar system operates with an 8 GHz bandwidth (18–26 GHz) and employs photonic frequency mixing for de-chirping radar echoes. Using a novel DCI method, the system achieved super-resolution imaging with a range resolution of 2 cm and azimuth and elevation resolutions of 0.3°. The radar’s unique design allows for enhanced forward-looking imaging, making it particularly useful for autonomous vehicles and complex 3D environments. Finally, in another 2024 study [37], the authors demonstrated a Microwave Photonic Cognitive Radar capable of subcentimeter resolution. This radar adaptively utilizes a wide spectral range for target detection and dynamic environmental monitoring. With a bandwidth of 22 GHz and an ADC sampling rate of 50 MSa/s, the radar achieved a range resolution of 0.73 cm. The system is designed for real-time imaging in congested spectral environments, offering significant advantages for autonomous driving and security surveillance.

## 3 Photonic radar principle

Photonic radar fundamentally differs from traditional radar systems by using light, rather than microwaves, to generate and process radar signals. At its core, photonic radar exploits the unique properties of photonics—such as large bandwidth and immunity to electromagnetic interference—to enhance detection capabilities. This section introduces the basic principles of photonic radar and outlines the key equations governing its operation. In photonic radar, an optical source, typically a laser, generates the radar signal. This signal is then modulated and transmitted as an optical carrier, which can be converted into an electrical signal at the receiver for processing. The principle of operation remains based on the radar’s time-of-flight measurement, where the time taken for a transmitted signal to reflect off a target and return to the receiver determines the distance to the target. The basic radar range equation used to calculate the distance R to a target in both traditional and photonic radar is **[**38–40**]**:


$$R=\frac{c\cdot \tau }{2}$$ (1)

where R is the distance to the target, *c* is the speed of light $\left(3\times 10^{-8}\;m/s\right)$,

In photonic radar, this time delay is measured using high-precision optical components, allowing for extremely accurate distance calculations, even for small or fast-moving targets.

### 3.1 FMCW radar principle

Photonic radar systems often employ FMCW techniques to measure both the distance and velocity of a target. In an FMCW radar, the transmitted signal is a frequency-modulated chirp. The frequency of the chirp signal is linearly increased over time. The difference in frequency between the transmitted and received signals, called the beat frequency, is used to determine the distance and velocity of the target.

The transmitted signal can be expressed as **[**38–40**]**:


$$s(t)=A\cos\left(2\pi \left(f_{c}t+\frac{B}{2T}t^{2}\right)\right)$$ (2)

Where A is the amplitude of the signal, $f_{c}$ is the carrier frequency, *B* is the bandwidth of the chirp signal, *T* is the duration of the chirp, and *t* is the time variable.

After reflection from a target at distance RRR, the received signal will be delayed by $\tau =\frac{2R}{c}$ and can be expressed as:


$$s_{r}(t)=A\cdot \cos\left(2\pi \left(f_{0}(t-\tau )+\frac{B}{2T}(t-\tau {)}^{2}\right)\right)$$ (3)

To extract the target information, the transmitted and received signals are mixed, resulting in a beat signal $s_{b}(t)$:


$$s_{b}(t)=s_{t}(t)\cdot s_{r}(t)$$ (4)

The frequency of the beat signal $f_{b}$ is related to the target’s distance R by:


$$f_{b}=\frac{B}{T}\cdot \frac{2R}{c}$$ (5)

This equation shows that the beat frequency $f_{b}\;$is proportional to the distance R of the target, allowing photonic radar systems to calculate the range precisely.

## 4 Proposed photonic radar modeling

**Fig 2:**
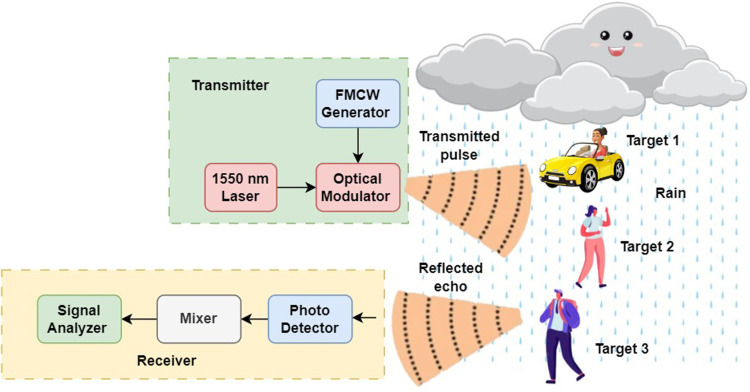
Proposed photonic radar for multiple target detections under atmospheric rain.

In the design and simulation of the proposed photonic radar system, as shown in Fig 2, we aim to assess its performance under various environmental conditions, beginning with ideal free-space propagation, followed by more complex scenarios involving atmospheric turbulence and rain-induced attenuation.

**Fig 3:**
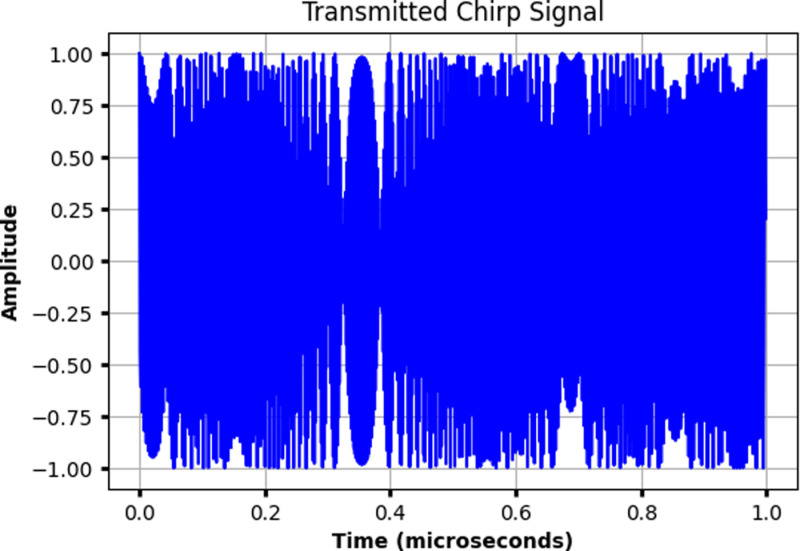
Transmitted chirp signal.

The radar system is designed around a stable optical signal source, typically a mode-locked or continuous-wave laser operating at a wavelength of 1550 nm. This wavelength is chosen for its minimal atmospheric absorption and compatibility with existing fiber-optic communication technologies. The system employs FMCW radar techniques, where a chirped signal as shown in Fig 3—linearly modulated in frequency over time—is used for precise range and velocity measurements.

In the initial phase of the simulation, a free-space model is used without considering attenuation. This allows us to model the basic operation of the photonic radar system in an ideal scenario. After propagating through free space and reflecting off objects, the radar antenna receives the reflected signal, which is then converted back into an electrical signal for processing. The received signal contains information about the target’s range and velocity, encoded as time delays and Doppler shifts. A beat frequency is generated by mixing the transmitted and received signals, and this beat frequency is processed using Fast Fourier Transform (FFT) techniques to extract precise range and velocity information. Following the free-space model, we incorporate Gamma-Gamma modeling to simulate the effects of atmospheric turbulence on the radar signal. The Gamma-Gamma distribution is widely used to model the intensity fluctuations of an optical wave propagating through a turbulent medium, representing the large-scale and small-scale effects of turbulence. In this phase of the simulation, the radar signal is subjected to the Gamma-Gamma channel model, which accounts for the random fluctuations in signal amplitude due to turbulence, providing a more realistic representation of radar performance in atmospheric conditions. Finally, we evaluate the impact of light and heavy rain on the radar system by applying a rain attenuation model. Rain causes significant attenuation of radar signals, particularly in the optical wavelength range. We model the rain-induced attenuation using specific attenuation values for both light rain (2 *mm/hr*) and heavy rain (25 *mm/hr*). These values are incorporated into the simulation to assess how rain affects the radar’s ability to detect and track targets at various distances. The radar’s performance under light and heavy rain conditions is compared against the free-space and turbulence-only scenarios to understand the degradation in signal strength and detection capabilities. For the simulation, we modeled three targets at distances of 50 *meters*, 100 *meters*, and 150 *meters*, respectively, and assumed a laser output power of 20 *dBm*, appropriate for typical autonomous vehicle scenarios. The simulations were carried out in Python, which allowed us to model the radar’s core components, including the chirped signal generation, signal transmission and reception, and the processing of the beat signal using FFT. By simulating the radar under free-space, turbulence, and rain-affected conditions, we were able to comprehensively evaluate the system’s performance across a range of realistic operating environments.

## 5 Mathematical modeling of free space and gamma gamma propagation

In this section, we outline the mathematical modeling of both free-space propagation and Gamma-Gamma channel modeling, which are key elements in the proposed photonic radar system.

### 5.1 Free-space propagation model

Free-space propagation is the simplest channel model, where the signal travels through the air without encountering significant obstacles or atmospheric effects. The received power in free-space propagation is governed by the Friis transmission equation, which describes the relationship between transmitted and received power over a distance in free space. The power received by the radar can be calculated as [41]:


$$P_{r}=P_{t}\cdot {\left(\frac{\lambda }{4\pi R}\right)}^{2}$$ (6)

where $P_{r}$ is the received power, $P_{t}\;$is the transmitted power, λ is the wavelength of the radar signal (in meters), R is the distance between the radar and the target (in meters),

The free-space path loss is given by:


$$L_{\text{fs}}={\left(\frac{4\pi R}{\lambda }\right)}^{2}$$ (7)

In a perfect free-space environment, there are no additional losses or attenuation factors, and the only decay in signal strength comes from the distance between the radar and the target. This model is essential for evaluating the basic performance of the radar in ideal conditions.

### 5.2 Gamma-gamma channel model for atmospheric turbulence

Atmospheric turbulence, caused by fluctuations in temperature and pressure, leads to random variations in the refractive index of the air, which in turn causes signal fading. These effects can be modeled using the Gamma-Gamma distribution, which captures the intensity fluctuations of an optical wave as it propagates through a turbulent medium. The Gamma-Gamma model is characterized by two shape parameters, α\alphaα and β\betaβ, which represent large-scale and small-scale turbulence, respectively. The probability density function (PDF) of the Gamma-Gamma distributed fading channel is given by [42–44]:


$$p(I)=\frac{2(\alpha \beta {)}^{\frac{\alpha +\beta }{2}}}{\Gamma (\alpha )\Gamma (\beta )}I^{\frac{\alpha +\beta }{2}-1}K_{\alpha -\beta }\left(2\sqrt{\alpha \beta I}\right)$$ (8)

where I is the intensity of the received signal, α is the large-scale turbulence parameter, β is the small-scale turbulence parameter, Γ (.) is the Gamma function and $K_{\alpha -\beta }$ is the modified Bessel function of the second kind.

In the Gamma-Gamma model, α and β are typically chosen based on the strength of the turbulence. Higher values of α and β represent stronger turbulence effects, leading to greater signal degradation. The received power under the Gamma-Gamma channel can be modeled as:


$$P_{r}=P_{t}\cdot {\left(\frac{\lambda }{4\pi R}\right)}^{2}\cdot \eta_{\text{GG}}$$ (9)

where $\eta_{\text{GG}}$ is a random variable representing the Gamma-Gamma fading process, distributed according to the PDF described above.

### 5.3 Rain attenuation model

In addition to turbulence, the radar signal is also affected by rain, which causes attenuation based on the specific rain rate (measured in mm/hr). The rain attenuation can be modeled as [45]:


$$A_{\text{rain}}=\alpha \cdot R^{\beta }$$ (10)

where $A_{\text{rain}}\;$is the attenuation due to rain (in dB/km), R is the rain rate (in mm/hr), α and β are empirical coefficients that depend on the frequency and rain characteristics.

The total attenuation due to rain over a distance ddd (in kilometers) is given by:


$$L_{\text{rain}}=A_{\text{rain}}\cdot d$$ (11)

Incorporating both the Gamma-Gamma fading and rain attenuation, the total received power at the radar receiver becomes:


$$P_{r}=P_{t}\cdot {\left(\frac{\lambda }{4\pi R}\right)}^{2}\cdot \eta_{\text{GG}}\cdot 10^{-\frac{L_{\text{rain}}}{10}}$$ (12)

This equation accounts for the combined effects of free-space path loss, turbulence, and rain attenuation on the radar signal.

## 6 Results and discussion

In this section, we will present the findings of the simulations and analyze the impact of various parameters, such as rain conditions (light rain and heavy rain), free-space propagation, and Gamma-Gamma fading models, on the performance of the photonic radar system. This section will compare the received power at different distances for each target and discuss the results in the context of autonomous vehicles.

**Fig 4:**
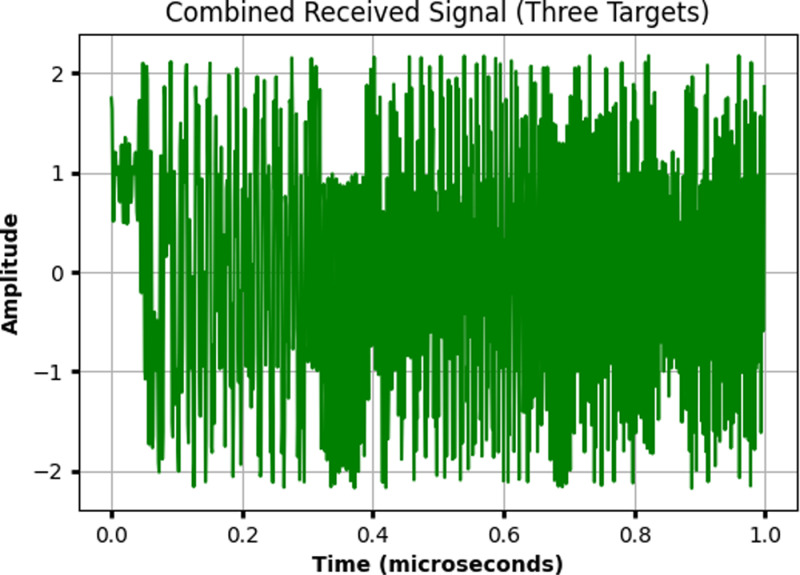
Combined received signal from three distinct targets under the free-space path loss model.

Fig 4 illustrates the combined received signal from three distinct targets under the free-space path loss model. The signal is measured over time, with the x-axis representing time in microseconds and the y-axis showing the amplitude of the received signal, ranging between -2 and 2. As the signals from each target are reflected and received by the radar system, they are superimposed, resulting in interference patterns due to the varying round-trip delays based on each target’s distance. The fluctuations in amplitude indicate the combined effect of the signals, which is a result of the constructive and destructive interference caused by the interaction of the delayed signals from each target. This plot highlights the complexity of isolating individual target responses in the presence of multiple reflections.

**Fig 5:**
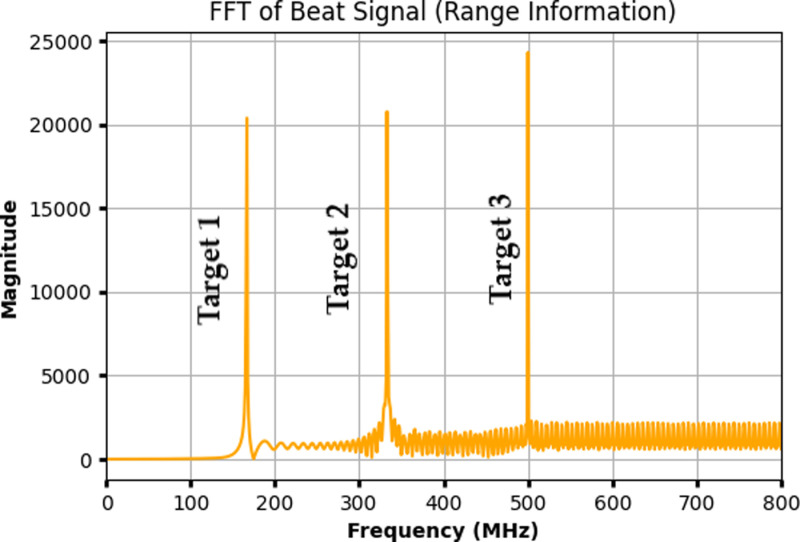
FFT of the beat signal showing range information for three distinct targets.

**Fig 6:**
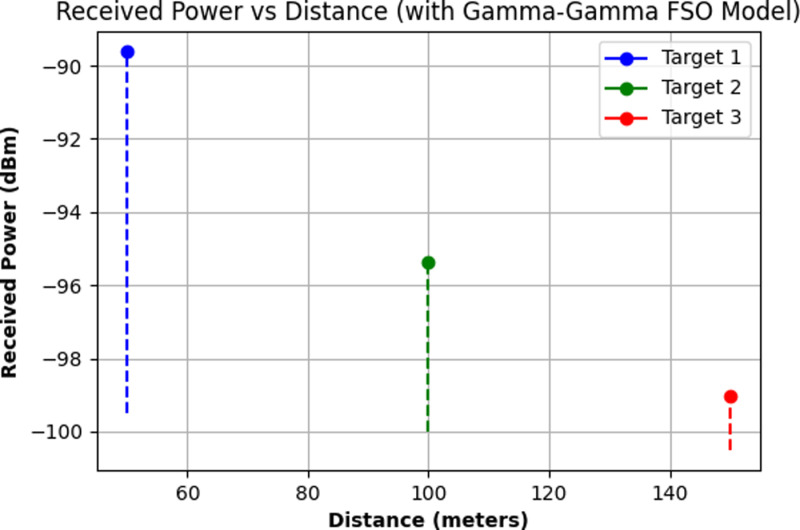
Received power vs. distance for three targets under the Gamma-Gamma FSO model.

Fig 5 shows the FFT of the beat signal representing the range information of three distinct targets. The x-axis represents frequency in megahertz (MHz), and the y-axis shows the magnitude of the FFT. The distinct peaks at specific frequencies indicate the presence of targets at different ranges. Each peak corresponds to the beat frequency generated by the difference in frequency between the transmitted chirp and the received signal reflected from each target. The height of the peaks signifies the strength of the reflections, while their position along the frequency axis corresponds to the round-trip delay, which is directly related to the distance of each target. The presence of three prominent peaks confirms the detection of three targets, each located at varying distances from the radar system. The spread of frequencies after the main peaks represents noise and other non-target reflections. Fig 6 shows the received power vs distance for three targets under the Gamma-Gamma FSO Model. The distances of the three targets are fixed at 50 meters, 100 meters, and 150 meters. The y-axis represents the received power in dBm, while the x-axis represents the target distances in meters. Each target is represented by a distinct color: Target 1 (blue), Target 2 (green), and Target 3 (red). The dashed lines indicate the attenuation of the received power as the distance increases, with the received power values decreasing as the targets are positioned farther from the radar. The use of the Gamma-Gamma model accounts for both large-scale and small-scale atmospheric turbulence, which affects the signal strength.

The graph highlights that Target 1, at 50 meters, has the highest received power of approximately -90 dBm, while Target 2 and Target 3, positioned farther away, exhibit lower received powers of around -96 dBm and -100 dBm, respectively. The attenuation follows the expected trend where increased distance results in lower received power due to free-space path loss and atmospheric turbulence effects.

**Fig 7:**
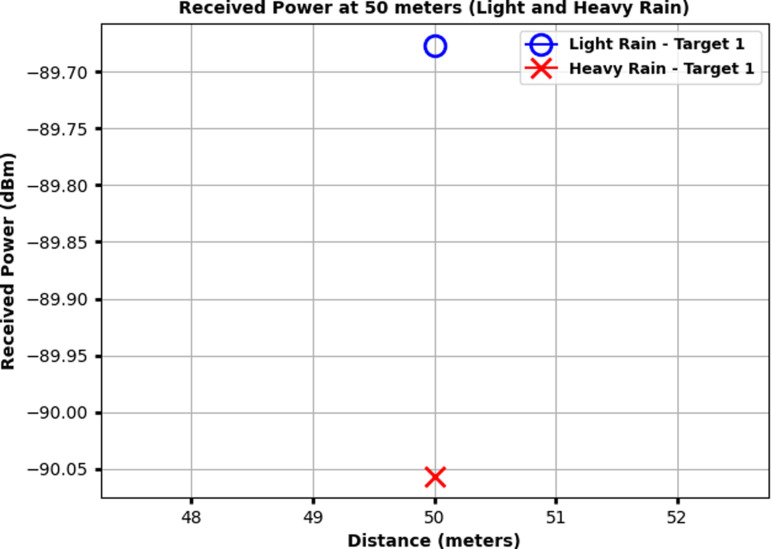
Received power at 50 meters for Target 1 under light rain and heavy rain conditions.

Fig 7 illustrates the received power at 50 meters for Target 1 under both light rain and heavy rain conditions. The x-axis represents the distance around 50 meters, while the y-axis shows the received power in dBm. The received power for light rain is marked by a blue circle, while the received power for heavy rain is indicated by a red cross. The graph demonstrates that the signal strength under heavy rain is noticeably lower compared to light rain. Specifically, for Target 1, the received power in light rain is approximately −89.70 dBm, while in heavy rain, it decreases to around −90.05 dBm. This difference highlights the significant impact of rain-induced attenuation on signal propagation, with heavier rain resulting in greater signal degradation.

**Fig 8:**
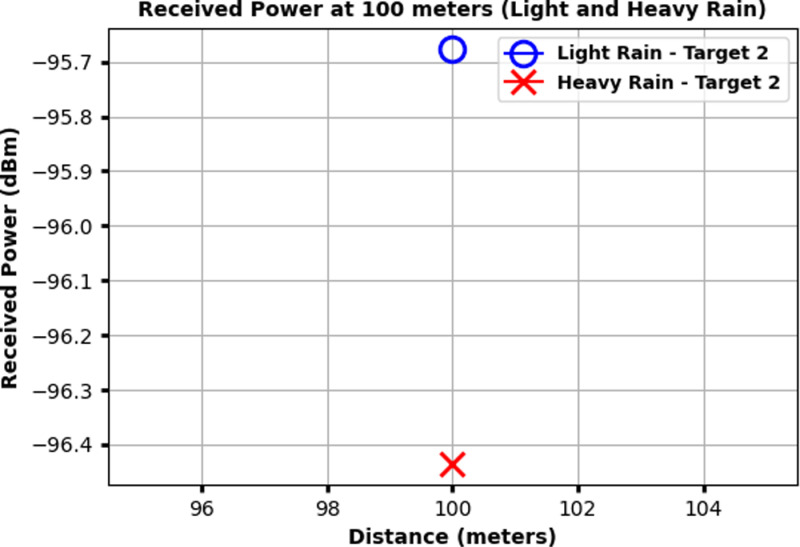
Received power at 100 meters for Target 2 under light rain and heavy rain conditions.

**Fig 9:**
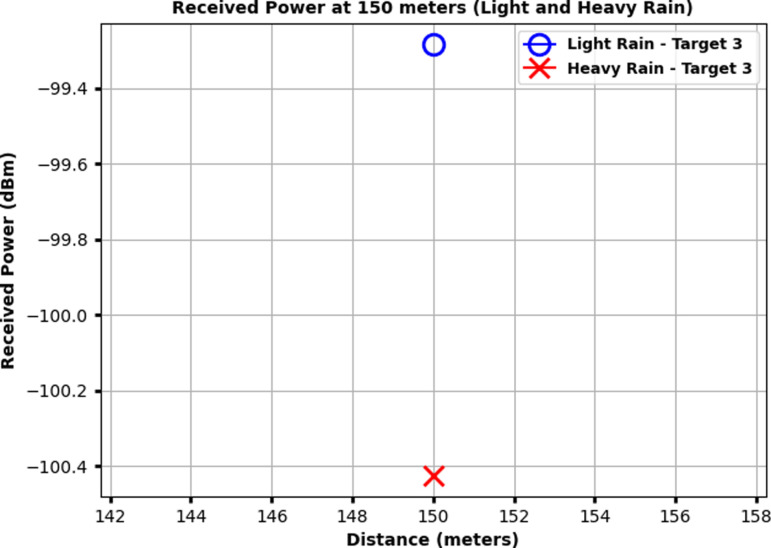
Received power at 150 meters for Target 3 under light rain and heavy rain conditions.

Fig 8 illustrates the received power at 100 meters for Target 2 under both light rain and heavy rain conditions. The x-axis represents the distance around 100 meters, and the y-axis shows the received power in dBm. The received power for light rain is indicated by a blue circle, while the received power for heavy rain is marked by a red cross. For Target 2, the received power in light rain is approximately Ȓ95.7 dBm, whereas in heavy rain, it drops to around −96.4 dBm. This decrease demonstrates the increased signal attenuation caused by heavy rain, which significantly reduces the received power compared to light rain conditions. Similarly, Fig 9 shows the received power at 150 meters for Target 3 under both light rain and heavy rain conditions. The x-axis represents the distance around 150 meters, and the y-axis depicts the received power in dBm. The received power for light rain is marked by a blue circle, while the received power for heavy rain is indicated by a red cross.

For Target 3, the received power in light rain is approximately -99.4 dBm, while under heavy rain, it decreases to about -100.4 dBm. This difference demonstrates the increased attenuation caused by heavy rain at longer distances, resulting in more significant signal degradation.

## 7 Conclusion

In this study, we modeled and simulated the performance of a photonic radar system under various environmental conditions, focusing on the impact of free-space path loss, Gamma-Gamma atmospheric turbulence, and rain attenuation (both light and heavy rain). Through our simulations, we were able to observe the distinct effects of these factors on the received signal power and the ability of the radar to detect targets at different distances. The results show that free-space path loss combined with Gamma-Gamma fading significantly affects the received power, particularly as the target distance increases. Atmospheric turbulence, modeled by the Gamma-Gamma fading channel, introduces variability in the signal, simulating real-world conditions where atmospheric disturbances can cause fluctuations in the radar’s performance. Furthermore, the rain attenuation models demonstrate the pronounced impact of rain on the radar system’s effectiveness. In light rain conditions, the signal experiences moderate attenuation, whereas in heavy rain, the received power is substantially reduced. As the target distance increases, the effect of heavy rain becomes more severe, highlighting the challenge of maintaining reliable radar performance in adverse weather conditions. This is particularly evident when comparing the received power across different targets under both light and heavy rain scenarios. While photonic radar offers significant advantages in terms of high-resolution sensing, immunity to electromagnetic interference, and enhanced performance in adverse weather conditions, certain challenges must be considered for its practical implementation. One key challenge is the manufacturing complexity associated with high-precision optical components, which can lead to increased costs. Additionally, seamless integration with existing autonomous vehicle sensor architectures requires further research to ensure compatibility with current signal processing frameworks and decision-making algorithms. Future work should focus on developing cost-effective fabrication techniques, improving integration strategies, and optimizing signal processing methods to enhance the scalability and feasibility of photonic radar in real-world applications. Future work should focus on extending the analysis by incorporating a larger number of simulation runs to evaluate the statistical variability of received power under different environmental conditions. Additionally, exploring the limits of environmental effects on photonic radar performance will provide deeper insights into the system’s resilience under diverse weather conditions. The study’s findings are critical for the application of photonic radar in scenarios such as autonomous vehicles, where reliable and accurate target detection is essential for safety. The degradation of radar performance due to rain and atmospheric conditions must be considered in the design and deployment of such systems. Future work could focus on adaptive techniques to mitigate the impact of environmental conditions or on improving the robustness of the radar system for use in harsh weather environments.
